# Public preferences regarding slow codes in critical care

**DOI:** 10.1111/bioe.13359

**Published:** 2024-10-03

**Authors:** Philipp Sprengholz

**Affiliations:** ^1^ Institute of Psychology University of Bamberg Bamberg Germany; ^2^ Institute for Planetary Health Behaviour University of Erfurt Erfurt Germany; ^3^ Implementation Science Bernhard Nocht Institute for Tropical Medicine Hamburg Germany

**Keywords:** critical care, empirical ethics, public opinion, slow code

## Abstract

The term *slow code* refers to an intentional reduction in the pace or intensity of resuscitative efforts during a medical emergency. This can be understood as an intermediate level between *full code* (full resuscitation efforts) and *no code* (no resuscitation efforts) and serves as a symbolic gesture when intervention is considered medically futile. While some previous research acknowledges the slow code as an integral part of clinical practice, many ethicists have condemned the practice as dishonest and causing unnecessary pain for the patient. As the public's views on this issue have been largely absent from the discussion to date, two vignette experiments were performed to investigate their perceptions. The findings indicate that laypersons believe that slow codes are commonplace and often prefer them over a no code. While a full code was perceived as the standard approach and rated most ethical and least punishable, the present results do not support the widespread assumption that laypersons generally oppose slow codes, and this finding should inform ethical discussion and clinical practice.

## 1 INTRODUCTION

The term *slow code* (or *show code*) refers to a deliberate reduction in the speed or intensity of resuscitative efforts during a cardiac arrest or other life‐threatening medical emergencies. This can be understood as an intermediate level between *full code* (full resuscitation efforts) and *no code* (no resuscitation efforts) and serves as a symbolic gesture when intervention is considered medically futile. Slow codes can take many forms, including briefer attempts to resuscitate, moving at a slower pace, or not attempting all interventions available. While slow codes are unlikely to benefit the patient, they may be of help to family members who feel unable to consent to a do‐not‐resuscitate order.^1^ Although many ethicists have condemned slow codes as dishonest and a cause of needless pain for the patient, previous research indicates that the practice is widespread. In one recent survey of U.S. medical professionals,^2^ about two‐thirds reported caring for a patient subject to a slow code. While a narrow majority (52%) of respondents perceived slow codes as ethical if treatment was medically futile, 19% favored a no code, and 28% favored a full code. Other evidence from Ireland^3^ and Israel^4^ confirms that slow codes form part of medical care in these countries as many nurses and doctors consider them ethical beneficent acts. These instances conflict with the weight of ethical opinion in the literature, but the views of the public are largely missing from the ongoing discussion. To fill this important gap in the literature, two survey experiments were performed to investigate laypersons' perceptions of slow codes. The findings should inform future ethical debate and can help to improve critical care by taking account of public preferences and perspectives.

## 2 EXPERIMENT 1

Experiment 1 investigated how members of the public evaluated the application of a full code, no code, or slow code in a medical emergency where the patient's family requested extensive treatment even though the chances of meaningful recovery were minimal.

### 2.1 Method

#### 2.1.1 Participants and design

On October 13, 2023, 450 U.S. residents were recruited via CloudResearch's Connect platform. The sample was quota‐representative for age, gender, race, and ethnicity according to the U.S. Census. Participants ranged in age from 18 to 80 years (*M* = 45.68, *SD* = 15.81); of these, 50% were male, 78% identified as White, and 14% identified as Black or African American. They received 0.30 dollars for completing the survey. The experiment employed a between‐participant design with three conditions. The sample size was set to 150 participants per condition in an a priori power analysis to be able to detect small effects (*d* = 0.2, *α* = 0.05, 1 – *β* = 0.80) when comparing the conditions.

#### 2.1.2 Procedure and materials

At the beginning of the experiment, participants were asked to read a short vignette about Mr. Johnson, a 70‐year‐old man with a history of heart disease, who had been admitted to the intensive care unit following a severe heart attack that left him in a critical condition (Table 1). They were also advised that although the medical team had conducted a thorough assessment and determined that Mr. Johnson's heart function was critically compromised, with minimal chance of recovery, his family had requested that everything possible be done to prolong his life. The vignette was informed by evidence showing that (a) patients admitted to the ICU are often of older age,^5^ (b) cardiac events are a common reason for ICU admission,^6^ and (c) ICU patients are frequently visited by loved ones^7^ who often demand maximum care. After reading the vignette, participants were randomly assigned to one of three conditions.

(1) In the *full code* condition, participants read that the medical team had decided to use all available and aggressive resuscitation measures to revive Mr. Johnson, including intubation, defibrillation, and drugs. They were informed that the treatment would be painful for Mr. Johnson and would not allow him to spend his remaining time peacefully with his family.
(2) In the *no code* condition, participants read that the medical team had decided against resuscitation and instead focused on palliative care to reduce Mr. Johnson's pain as much as possible; this also allowed him to spend his remaining time peacefully with his family.
(3) In the *slow code* condition, participants read that the medical team had decided to attempt resuscitation but at a slower pace and with less intensity. This served to honor the family's wishes while also recognizing the medical reality and seeking to prevent pain for Mr. Johnson in his remaining time.

**Table 1:** Experimental vignettes.

Full code condition	No code condition	Slow code condition
Mr. Johnson is a 70‐year‐old man with a history of heart disease and diabetes. He has been admitted to the ICU due to a severe heart attack, which has left him in a critical condition. Mr. Johnson is struggling to breathe. The medical team has conducted a thorough assessment and determined that his heart function is critically compromised, and there's minimal chance of a meaningful recovery. However, his family requests that everything possible is done to prolong his life.		
The medical team decides for a “full code.” This means that they use all available and aggressive resuscitation measures, including intubation, defibrillation and drugs, in an attempt to revive Mr Johnson. This will be painful for Mr Johnson and, in the likely event that he does not recover, it also means that he will not be able to spend his remaining time peacefully with his family.	The medical team decides for a “no code.” This means that they do not use aggressive resuscitation measures and instead focus on palliative care to reduce Mr Johnson's pain as much as possible. This decision allows Mr Johnson to spend his remaining time peacefully with his family.	The medical team decides for a “slow code.” This means that they carry out resuscitation efforts, but at a slower pace or with less intensity, recognizing that Mr Johnson's condition is unlikely to improve. This approach honors the family's wishes while recognizing medical reality and avoiding pain for Mr Johnson in his remaining time.

After reading the text, participants in each condition were asked to respond to each of the following four statements on a 7‐point scale ranging from *strongly disagree* to *strongly agree* (only the endpoints were labeled). (1) *The medical team's decision is in Mr. Johnson's best interests*. (2) *The medical team's decision is in the best interests of Mr. Johnson's family*. (3) *The medical team's decision is ethical*. (4) *The medical team should be punished for their decision*. In addition, participants were asked to indicate how often medical teams typically make the presented decision in such situations (on a 7‐point scale ranging from *very seldom* to *very often*; only the endpoints were labeled). Original vignettes and measures are provided in the online supplement.

### 2.2 Results

**Figure 1:**
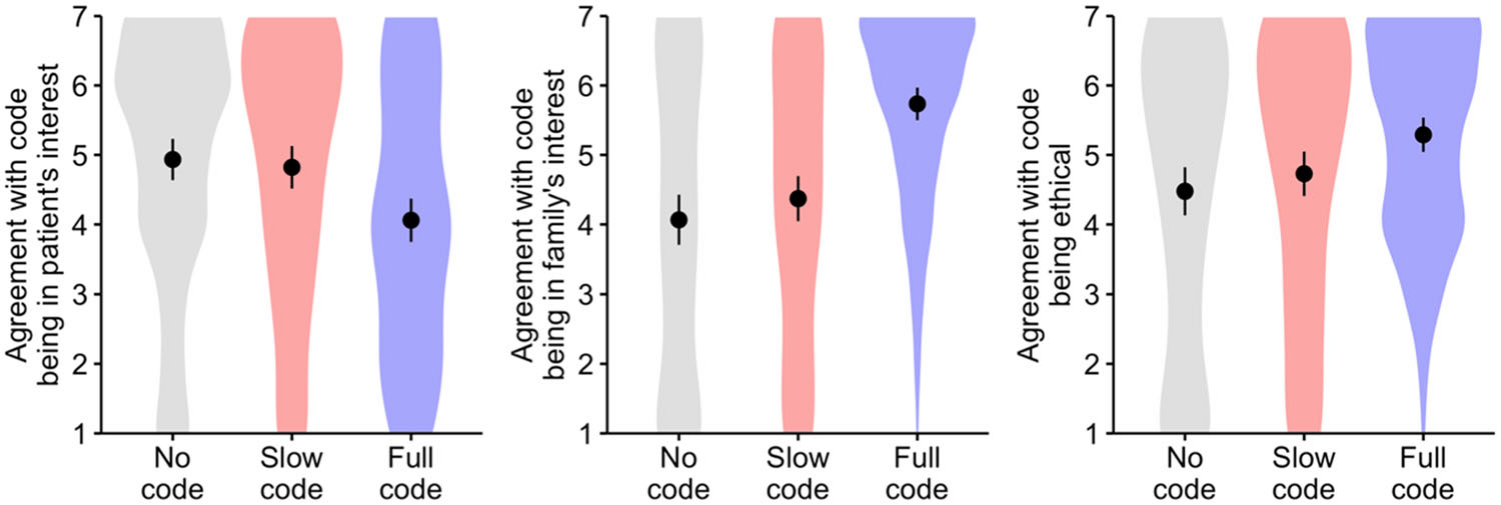
Perception of no code, slow code, and full code decisions in Experiment 1. Participants ratings of full code (*n* = 148), no code (*n* = 151), or slow code (*n* = 151) decisions; color bands denote answer distributions; dots denote mean values, and bars indicate 95% confidence intervals.

Analyses were performed using R (version 4.3.1) using a significance level of *α* = 0.05. Welch's *t*‐tests and Cohen's *d* were calculated to compare participant's ratings between the three vignettes. As shown in Figure 1, the no code and slow code scenarios attracted similar ratings, but the full code decision was perceived to be less aligned with the patient's interests (mean *M* = 4.06, standard deviation *SD* = 1.92) than the no code (*M* = 4.93, *SD* = 1.85, *d* = 0.46, *p* < 0.001) and slow code decisions (*M* = 4.82, *SD* = 1.91, *d* = 0.40, *p* < 0.001). Conversely, participants in the full code condition agreed more strongly that this decision aligned with the family's interests (*M* = 5.74, *SD* = 1.44) compared to participants in the no code (*M* = 4.07, *SD* = 2.24, *d* = 0.89, *p* < 0.001) and slow code conditions (*M* = 4.37, *SD* = 2.02, *d* = 0.78, *p* < 0.001). The full code decision was also rated as slightly more ethical (*M* = 5.29, *SD* = 1.52) than the no code (*M* = 4.48, *SD* = 2.15, *d* = 0.43, *p* < 0.001) and slow code (*M* = 4.73, *SD* = 1.99, *d* = 0.31, *p* = 0.006) decisions. Across all three evaluations, no significant differences were found between the no code and slow code conditions (*p* > 0.215).

To investigate whether ethicality ratings differed by age and gender, linear regression analyses were performed for each experimental condition. Compared to younger participants, older individuals perceived no code decisions as less ethical (*b* = −0.03, *SE* = 0.01, *p* = 0.008) and full code decisions as more ethical (*b* = 0.02, *SE* = 0.01, *p* = 0.009). However, perceived ethicality of slow code decisions did not differ significantly by age (*b* = −0.01, *SE* = 0.01, *p* = 0.246). Women tended to rate all decisions as less ethical, but this difference was significant only in the full code condition (*b* = −0.61, *SE* = 0.25, *p* = 0.014).

**Figure 2:**
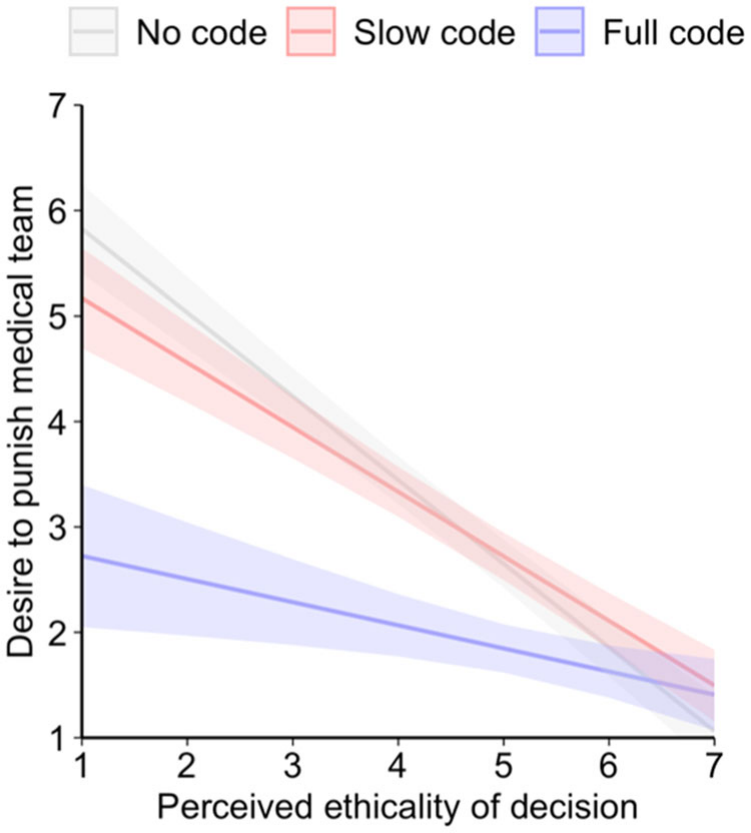
Desire to punish in Experiment 1. Results from linear regression analysis regressing the desire to punish a medical team on its decision, the perceived ethicality of this decision, and their interaction, ribbons indicate 95% confidence intervals; *R* ^2^ = 0.49.

Average desire to punish the medical team for their decision was low in all three conditions but lower in the full code decision (*M* = 1.78, *SD* = 1.35) compared to the no code (*M* = 3.07, *SD* = 2.20, *d* = 0.70, *p* < 0.001) and slow code (*M* = 2.89, *SD* = 1.90, *d* = 0.67, *p* < 0.001) decisions. In the case of the latter two, there was no difference in desire to punish (*p* = 0.451). Regressing punishment desire on the experimental conditions, their perceived ethicality, and the interaction revealed a moderating effect. As shown in Figure 2, no code and slow code decisions strongly increased punishment desire when the ethicality of these decisions was perceived as low. However, for full code decisions, low ethicality ratings merely increased punishment desire.

When asked how often medical teams in such situations were likely to make a decision like the one presented in their experimental condition, participants assumed that this was frequently the case. While the full code decision was perceived as more common (*M* = 5.78, *SD* = 1.38) than no code (*M* = 5.01, *SD* = 1.76, *d* = 0.49, *p* < 0.001) and slow code (*M* = 4.87, *SD* = 1.53, *d* = 0.63, *p* < 0.001) decisions, participants did not perceive the latter two as rare occurrences.

### 2.3 Discussion

The first experiment investigated laypersons' perceptions of full code, no code, and slow code decisions where there was minimal chance of recovery, but the family requested that everything possible should be done to save the patient's life. Of the three decisions, full code was perceived as more ethical and better aligned with the family's wishes but not with the patient's interests. Furthermore, full code decisions were perceived as more frequent in everyday practice and were associated with a lower desire to punish the medical team—even when the decision was considered unethical. While this suggests that the public favors full code decisions, slow codes (which are often condemned in the literature) were evaluated more positively than expected, as most participants tended to agree that they are ethical. While older participants perceived full code (no code) as more (less) ethical, ethicality ratings of the slow code did not differ by age. This may reflect the middle ground nature of slow codes which appear to respect family wishes and perhaps reduce stress and anxiety among close relatives (which may be the focus of older adults who have been shown to be more altruistic than younger ones)^8^ while at the same time limiting patient pain (which may be the focus of younger adults).

Interestingly, evaluations of slow code and no code decisions did not differ significantly in terms of ethicality, alignment with patient and family interests, punishment desire, or expected frequency in daily practice. This suggests that laypersons do not differentiate between slow codes and no codes, perhaps because a slow code is simply understood as a (deceptive) form of no code. However, as participants only read about one specific condition, evaluations might differ if people were just asked to compare no codes and slow codes. Furthermore, the results relate to a scenario in which the family asked for extensive treatment; if no such information was provided, ratings of slow codes may have been lower. To further investigate whether no codes and slow codes are perceived as interchangeable, a second experiment was performed.

## 3 EXPERIMENT 2

In Experiment 2, participants were asked to compare no code and slow code decisions, and the family's request was manipulated to investigate the effect on laypersons' treatment preferences.

### 3.1 Method

#### 3.1.1 Participants

On October 15, 2023, 451 U.S. residents were recruited via CloudResearch's Connect platform. The sample was quota‐representative for age, gender, race, and ethnicity according to the U.S. Census. Participants were between 18 and 85 years old (*M* = 45.58, *SD* = 15.46), 50% were male, 78% identified as white, and 14% identified as Black or African American. They received 0.30 dollars for completion of the survey.

#### 3.1.2 Procedure and materials

At the beginning of the experiment, participants again read the Mr. Johnson vignette. This time, however, they were asked to imagine that the medical team had to decide between just two options: a no code or a slow code (as defined in Experiment 1). Additionally, the family's wishes were manipulated; about half of the sample read that Mr. Johnson's family had requested that everything possible should be done to prolong his life, while the others received no information about the family's preferences.

After reading the text, participants were asked to indicate which of these two options aligned more with Mr. Johnson's interests; which option aligned more with his family's interests; which option was more ethical; and which option the medical team should choose. For all but the final question, participants could indicate that neither option was better. The order of choice options was randomized for each measure. Original vignettes and measures are provided in the online supplement.

### 3.2 Results

Analyses were performed using R (version 4.3.1) using a significance level of α = 0.05. As shown in Table 2, most participants perceived the no code option as better aligned with the patient's interests than the slow code option, and the two experimental groups did not differ significantly in this regard. While most participants perceived the slow code option as better aligned with family interests, this effect was stronger when participants were advised that the family had requested that everything possible be done (80% vs. 70%; *χ* ^2^ (1, 451) = 5.66, *p* = 0.017). When asked which option they considered more ethical, about a third favored no code, a third favored slow code, and a third rated both as equally ethical; this distribution did not differ significantly between experimental conditions. The majority felt that the medical team should choose a slow code approach. However, this fell from 69% when the family requested that everything possible be done to 59% when no information was provided about the family's preferences.

**Table 2:** Choice frequencies in Experiment 2.

	Family request (*n* = 225)			No family request (*n* = 226)			*χ* ^2^	*p*
	No code	Slow code	Neither	No code	Slow code	Neither		
More in patient's interest	49%	23%	28%	43%	31%	26%	3.67	0.160
More in family's interest	8%	80%	11%	15%	70%	15%	6.56	0.038
More ethical	32%	35%	33%	31%	33%	35%	0.27	0.876
Preferred choice	31%	69%		41%	59%		4.94	0.026

*Note*: Shares of participants deciding if a no code or slow code or neither is more in patient and family interests, more ethical, and their preferred choice, depending on experimental manipulation (reading or not reading about the patient's family requesting everything possible be done to save his life). Missing percentages reflect rounding to whole numbers. *χ* ^2^ scores indicate significant differences between shares in the two experimental conditions.

There was an association between perceiving an option as ethical and choosing that option. Of those who perceived no code as more ethical, 74% favored that approach; of those who perceived slow code as more ethical, 97% favored that approach. It is worth noting that among those who rated both options as equally ethical, a majority (67%) favored the slow code option.

### 3.3 Discussion

The second experiment investigated public preferences when a medical team had to decide between no code and slow code. The results suggest that most laypersons are likely to express a clear preference for one option or the other; only a third regard both options as equally ethical, indicating that the two are not perceived as interchangeable. The slow code preference strengthened when participants were informed that the patient's family had requested maximum intervention. Overall, these results contrast starkly with the common expectation that the public disapproves of slow codes.

## 4 CONCLUSION

Previous research indicates that slow codes are an integral part of clinical practice.^9^ While many ethicists have condemned slow codes as dishonest and a source of unnecessary pain for the patient,^10^ others have argued that this approach may be of help to family members who feel unable to consent to a do‐not‐resuscitate order.^11^ The findings of the two experiments reported here indicate that laypersons believe that slow codes are introduced on a regular basis and that a majority perceive this approach as more or equally ethical when compared to a no code decision. While full code interventions seem to be perceived as the standard and are rated most ethical and least punishable, the present findings conflict with the widespread belief that the public opposes slow codes. This may reflect the middle‐ground status of slow codes; while Experiment 1 found that laypersons perceive slow code as better aligned with patient interests than full code, Experiment 2 identified a preference for slow code rather than no code with regard to family interests—even in the absence of any information about family preferences. This may explain why a majority favored slow code rather than no code when required to choose between the two.

The presented experiments offer some important insights into public opinion and preferences. However, the results should be interpreted and generalized with care, as they refer to a specific scenario, and laypersons' responses might differ in other cases—for instance, if the patient was younger, or information was provided about an advance directive, or the family had asked the medical team to focus exclusively on palliative care rather than resuscitation. The present findings also reflect the views of some but not all U.S. residents. While the samples were diverse, people without internet access could not participate and opinions may differ for old individuals as well as people living in other countries. Future research should investigate public perceptions in more diverse groups. It is especially important to better understand the reasons behind individual differences. For instance, previous experiences, the level of education, or moral values may be linked to the support for slow codes. Such potential correlates have not been assessed in the two experiments presented here and future research should take them into account. Nevertheless, the findings here make a valuable contribution to the ongoing discussion of slow codes. Despite many ethicists' opposition, the wider public seems to view the practice more positively than might be expected. Clearly, both scholars and laypersons may favor recently proposed alternatives such as informed non‐dissent (i.e., allowing the physician to bear the major burden of decision making while ensuring that families retain the power to override any such decision)^12^ and the transparent use of slow codes^13^ rather than the classical approach, and this seems a promising avenue for future research and clinical practice.

## ETHICS STATEMENT

The experiments were conducted in accordance with German Psychological Association guidelines. Ethical clearance was obtained from the University of Bamberg's institutional review board (#2023‐10/39), and all participants provided informed consent to use and share their data for scientific purposes without disclosure of their identities.

## Supporting information

Supporting information.

## Footnotes

^1^
Lantos, J. D., & Meadow, W. L. (2011). Should the “slow code” be resuscitated? *The American Journal of Bioethics*, *11*(11), 8–12. https://doi.org/10.1080/15265161.2011.603793; Gazelle, G. (1998). The slow code—Should anyone rush to its defense? *New England Journal of Medicine*, *338*(7), 467–469. https://doi.org/10.1056/NEJM199802123380712; Muller, J. H. (1992). Shades of blue: The negotiation of limited codes by medical residents. *Social Science & Medicine*, *34*(8), 885–898. https://doi.org/10.1016/0277-9536(92)90257-Q; Weinacker, A. (2011). The “slow code” should be a “no code.” *The American Journal of Bioethics*, *11*(11), 27–29. https://doi.org/10.1080/15265161.2011.608244

^2^
Piscitello, G. M., Kapania, E. M., Miller, W. D., Rojas, J. C., Siegler, M., & Parker, W. F. (2020). Variation in ventilator allocation guidelines by us state during the coronavirus disease 2019 pandemic: A systematic review. *JAMA Network Open*, *3*(6), e2012606. https://doi.org/10.1001/jamanetworkopen.2020.12606

^3^
Kelly, J. (2008). Nurses' and doctors' perspectives on slow codes. *Nursing Ethics*, *15*(1), 110–120. https://doi.org/10.1177/0969733007084164

^4^
Ganz, F. D., Sharfi, R., Kaufman, N., & Einav, S. (2019). Perceptions of slow codes by nurses working on internal medicine wards. *Nursing Ethics*, *26*(6), 1734–1743. https://doi.org/10.1177/0969733018783222

^5^
Creagh‐Brown, B., & Green, S. (2014). Increasing age of patients admitted to intensive care, and association between increased age and greater risk of post‐ICU death. *Critical Care*, *18*(S1), P56, cc13246. https://doi.org/10.1186/cc13246

^6^
Jeon, E. J., Lee, H. M., Cho, S. G., Kang, H. K., Kwak, H. W., Song, J. H., Jung, J. W., Choi, J. C., Shin, J. W., Park, I. W., Choi, B. W., & Kim, J. Y. (2008). Causes of fever in the ICU ‐ A prospective, cohort study. *The Korean Journal of Critical Care Medicine*, *23*(1), 13. https://doi.org/10.4266/kjccm.2008.23.1.13

^7^
Eriksson, T., & Bergbom, I. (2007). Visits to intensive care unit patients—Frequency, duration and impact on outcome. *Nursing in Critical Care*, *12*(1), 20–26. https://doi.org/10.1111/j.1478-5153.2006.00196.x

^8^
Sparrow, E. P., Swirsky, L. T., Kudus, F., & Spaniol, J. (2021). Aging and altruism: A meta‐analysis. *Psychology and Aging*, *36*(1), 49–56. https://doi.org/10.1037/pag0000447

^9^
Piscitello, E. M., et al., op. cit. note 2.

^10^
Gazelle, op. cit. note 1; Muller, op. cit. note 1; Weinacker, op. cit. note 1.

^11^
Lantos & Meadow, op. cit. note 1.

^12^
Kon, A. A. (2011). Informed non‐dissent: A better option than slow codes when families cannot bear to say “let her die.” *The American Journal of Bioethics*, *11*(11), 22–23. https://doi.org/10.1080/15265161.2011.603796

^13^
Forman, E. N., & Ladd, R., E. (2012). Why not a slow code? *AMA Journal of Ethics*, *14*(10), 759–762. https://doi.org/10.1001/virtualmentor.2012.14.10.ecas1-1210

