# Supplementary material for: Public preferences regarding slow codes in critical care
Source: Bioethics. 2024 Oct 3;39(4):337–42. doi: 10.1111/bioe.13359 (PMC12015798; doi:10.1111/bioe.13359)
Supplement: Supplementary file 1 — Supporting information. [file BIOE-39-337-s001.zip › osfstorage-archive.zip!/Experiment 2/questionnaire.pdf]

Fragebogen

1 Intro

Trigger:

Zufallstrigger - Group - Bedingung: - Ausführungsposition: "direkt"
c_0001 = BALANCED RAND (1 , 2)

Welcome!

This survey is conducted by researchers of the University of Bamberg (Germany). Participation will take about 2 minutes. You will be asked to carefully read a short text about a situation in a hospital and answer a few questions about it.

All of your answers will be anonymized and used for scientific purposes only. Participation is voluntary and can be stopped at any time. In case of questions, please contact philipp.sprengholz@uni-bamberg.de.

Click next if you agree with the above terms and want to start the survey.

2 Scenario

Please read the following text very carefully. Take your time, there is no need to rush.

Mr. Johnson is a 70-year-old man with a history of heart disease and diabetes. He has been admitted to the ICU due to a severe heart attack, which has left him in a critical condition. Mr. Johnson is struggling to breathe. The medical team has conducted a thorough assessment and determined that his heart function is critically compromised, and there's minimal chance of a meaningful recovery.

The team now has the choice between two options:

Option 1: No code. If the medical team decides for a “no code”, this means that they do not use aggressive resuscitation measures and instead focus on palliative care to reduce Mr Johnson's pain as much as possible. This decision allows Mr Johnson to spend his remaining time peacefully with his family.

Option 2: Slow code. If the medical team decides for a “slow code”, this means that they carry out resuscitation efforts, but at a slower pace or with less intensity, recognising that Mr Johnson's condition is unlikely to improve. This approach honors the family's wishes while recognising medical reality and avoiding pain for Mr Johnson in his remaining time.

Please read the following text very carefully. Take your time, there is no need to rush.

Mr. Johnson is a 70-year-old man with a history of heart disease and diabetes. He has been admitted to the ICU due to a severe heart attack, which has left him in a critical condition. Mr. Johnson is struggling to breathe. The medical team has conducted a thorough assessment and determined that his heart function is critically compromised, and there's minimal chance of a meaningful recovery. However, his family requests that everything possible is done to prolong his life.

The team now has the choice between two options:

Option 1: No code. If the medical team decides for a “no code”, this means that they do not use aggressive resuscitation measures and instead focus on palliative care to reduce Mr Johnson's pain as much as possible. This decision allows Mr Johnson to spend his remaining time peacefully with his family.

Option 2: Slow code. If the medical team decides for a “slow code”, this means that they carry out resuscitation efforts, but at a slower pace or with less intensity, recognising that Mr Johnson's condition is unlikely to improve. This approach honors the family's wishes while recognising medical reality and avoiding pain for Mr Johnson in his remaining time.

What do you think about the two options? Please answer the following questions.

Which option is more honest?

- ◯ The no code
- ◯ The slow code
- ◯ Neither option is more honest than the other

Which option is more in Mr Jonson's interests?

- ◯ The no code
- ◯ The slow code
- ◯ Neither option is more in Mr Johnson’s interests than the other

Which option is more in the interests of Mr Johnson's family?

- ◯ The no code
- ◯ The slow code
- ◯ Neither option is more in the interests of Mr Johnson’s family than the other

Which option is more ethical?

- ◯ The no code
- ◯ The slow code
- ◯ Neither option is more ethical than the other

Which option should the medical team choose?

- ◯ The no code
- ◯ The slow code

Did Mr Johnson's family request that everything possible is done to prolong his life?

- ◯ Yes
- ◯ No

3 Endseite

Thank you for your participation! Please click on the link below to return to the CONNECT platform:

https://connect.cloudresearch.com/participant/project/7ba1b05312f04676b6c6a08b33a26515/complete
