# Supplementary material for: Public preferences regarding slow codes in critical care
Source: Bioethics. 2024 Oct 3;39(4):337–42. doi: 10.1111/bioe.13359 (PMC12015798; doi:10.1111/bioe.13359)
Supplement: Supplementary file 1 — Supporting information. [file BIOE-39-337-s001.zip › osfstorage-archive.zip!/Experiment 2/analysis_output.html]

Slow Code Experiment 2


# Slow Code Experiment 2

```
library(ggpubr)
```

```
## Loading required package: ggplot2
```

```
library(sjPlot)
library(ggsignif)

#platform_data <- read.csv("assignments.csv")
#data <- readxl::read_xlsx("data.xlsx")
#data$ParticipantId <- data$p_0001
#data[data == -77] <- NA
#data <- merge(data, platform_data, by = "ParticipantId", all.x = TRUE)
#writexl::write_xlsx(data, "data_experiment_2.xlsx")

data <- readxl::read_xlsx("data_experiment_2.xlsx")

data$CONDITION <- ifelse(data$c_0001 == 1, "no family request", "family request")

table(data$CONDITION)
```

```
## 
##    family request no family request 
##               225               226
```

# 1 Demographics

## 1.1 Age

```
psych::describe(data$Age)
```

```
##    vars   n  mean    sd median trimmed   mad min max range skew kurtosis   se
## X1    1 451 45.58 15.46     46   45.37 20.76  18  85    67 0.09     -1.2 0.73
```

## 1.2 Gender

```
table(data$Gender)
```

```
## 
##   Man Woman 
##   226   225
```

# 2 Manipulation check

```
data$CHECK_R <- ifelse(data$CHECK == 1, "classified as family request", "classified as no family request")
table(data$CONDITION, data$CHECK_R)
```

```
##                    
##                     classified as family request
##   family request                             218
##   no family request                          109
##                    
##                     classified as no family request
##   family request                                  7
##   no family request                             117
```

# 3 Evaluation of the codes

```
chisq.test(table(data$EVAL_PATIENT, data$CONDITION))
```

```
## 
##  Pearson's Chi-squared test
## 
## data:  table(data$EVAL_PATIENT, data$CONDITION)
## X-squared = 3.6702, df = 2, p-value = 0.1596
```

```
counts <- as.data.frame(table(data$EVAL_PATIENT, data$CONDITION))
counts
```

```
##   Var1              Var2 Freq
## 1    1    family request  111
## 2    2    family request   52
## 3    3    family request   62
## 4    1 no family request   97
## 5    2 no family request   70
## 6    3 no family request   59
```

```
prop.test(x = c(counts[4,]$Freq, counts[1,]$Freq), n = c(226, 225))
```

```
## 
##  2-sample test for equality of proportions with continuity correction
## 
## data:  c(counts[4, ]$Freq, counts[1, ]$Freq) out of c(226, 225)
## X-squared = 1.6169, df = 1, p-value = 0.2035
## alternative hypothesis: two.sided
## 95 percent confidence interval:
##  -0.1603889  0.0321293
## sample estimates:
##    prop 1    prop 2 
## 0.4292035 0.4933333
```

```
prop.test(x = c(counts[5,]$Freq, counts[2,]$Freq), n = c(226, 225))
```

```
## 
##  2-sample test for equality of proportions with continuity correction
## 
## data:  c(counts[5, ]$Freq, counts[2, ]$Freq) out of c(226, 225)
## X-squared = 3.1448, df = 1, p-value = 0.07617
## alternative hypothesis: two.sided
## 95 percent confidence interval:
##  -0.007468723  0.164715527
## sample estimates:
##    prop 1    prop 2 
## 0.3097345 0.2311111
```

```
prop.test(x = c(counts[6,]$Freq, counts[3,]$Freq), n = c(226, 225))
```

```
## 
##  2-sample test for equality of proportions with continuity correction
## 
## data:  c(counts[6, ]$Freq, counts[3, ]$Freq) out of c(226, 225)
## X-squared = 0.058114, df = 1, p-value = 0.8095
## alternative hypothesis: two.sided
## 95 percent confidence interval:
##  -0.10070356  0.07171635
## sample estimates:
##    prop 1    prop 2 
## 0.2610619 0.2755556
```

```
percents <- as.data.frame(prop.table(table(data$EVAL_PATIENT, data$CONDITION), margin = 2))
ggbarplot(
  data = counts,
  x = "Var2",
  y = "Freq",
  title = "More in patient interest",
  fill = "Var1",
  position = position_dodge(0.8)
) + scale_fill_manual(
  labels = c(
    "1" = "No code",
    "2" = "Show code",
    "3" = "Neither"
  ),
  values = c("red", "blue", "grey")
)
```

```
chisq.test(table(data$EVAL_FAMILY, data$CONDITION))
```

```
## 
##  Pearson's Chi-squared test
## 
## data:  table(data$EVAL_FAMILY, data$CONDITION)
## X-squared = 6.5635, df = 2, p-value = 0.03756
```

```
counts <- as.data.frame(table(data$EVAL_FAMILY, data$CONDITION))
counts
```

```
##   Var1              Var2 Freq
## 1    1    family request   19
## 2    2    family request  181
## 3    3    family request   25
## 4    1 no family request   33
## 5    2 no family request  159
## 6    3 no family request   34
```

```
prop.test(x = c(counts[4,]$Freq, counts[1,]$Freq), n = c(226, 225))
```

```
## 
##  2-sample test for equality of proportions with continuity correction
## 
## data:  c(counts[4, ]$Freq, counts[1, ]$Freq) out of c(226, 225)
## X-squared = 3.6087, df = 1, p-value = 0.05748
## alternative hypothesis: two.sided
## 95 percent confidence interval:
##  -0.001508853  0.124655362
## sample estimates:
##     prop 1     prop 2 
## 0.14601770 0.08444444
```

```
prop.test(x = c(counts[5,]$Freq, counts[2,]$Freq), n = c(226, 225))
```

```
## 
##  2-sample test for equality of proportions with continuity correction
## 
## data:  c(counts[5, ]$Freq, counts[2, ]$Freq) out of c(226, 225)
## X-squared = 5.6552, df = 1, p-value = 0.0174
## alternative hypothesis: two.sided
## 95 percent confidence interval:
##  -0.18427627 -0.01753298
## sample estimates:
##    prop 1    prop 2 
## 0.7035398 0.8044444
```

```
prop.test(x = c(counts[6,]$Freq, counts[3,]$Freq), n = c(226, 225))
```

```
## 
##  2-sample test for equality of proportions with continuity correction
## 
## data:  c(counts[6, ]$Freq, counts[3, ]$Freq) out of c(226, 225)
## X-squared = 1.2075, df = 1, p-value = 0.2718
## alternative hypothesis: two.sided
## 95 percent confidence interval:
##  -0.02722163  0.10588436
## sample estimates:
##    prop 1    prop 2 
## 0.1504425 0.1111111
```

```
percents <- as.data.frame(prop.table(table(data$EVAL_FAMILY, data$CONDITION), margin = 2))
ggbarplot(
  data = counts,
  x = "Var2",
  y = "Freq",
  title = "More in family interest",
  fill = "Var1",
  position = position_dodge(0.8)
) + scale_fill_manual(
  labels = c(
    "1" = "No code",
    "2" = "Show code",
    "3" = "Neither"
  ),
  values = c("red", "blue", "grey")
)
```

```
chisq.test(table(data$EVAL_ETHICAL, data$CONDITION))
```

```
## 
##  Pearson's Chi-squared test
## 
## data:  table(data$EVAL_ETHICAL, data$CONDITION)
## X-squared = 0.26508, df = 2, p-value = 0.8759
```

```
counts <- as.data.frame(table(data$EVAL_ETHICAL, data$CONDITION))
counts
```

```
##   Var1              Var2 Freq
## 1    1    family request   72
## 2    2    family request   79
## 3    3    family request   74
## 4    1 no family request   72
## 5    2 no family request   75
## 6    3 no family request   79
```

```
prop.test(x = c(counts[4,]$Freq, counts[1,]$Freq), n = c(226, 225))
```

```
## 
##  2-sample test for equality of proportions with continuity correction
## 
## data:  c(counts[4, ]$Freq, counts[1, ]$Freq) out of c(226, 225)
## X-squared = 7.6505e-31, df = 1, p-value = 1
## alternative hypothesis: two.sided
## 95 percent confidence interval:
##  -0.08888474  0.08605288
## sample estimates:
##    prop 1    prop 2 
## 0.3185841 0.3200000
```

```
prop.test(x = c(counts[5,]$Freq, counts[2,]$Freq), n = c(226, 225))
```

```
## 
##  2-sample test for equality of proportions with continuity correction
## 
## data:  c(counts[5, ]$Freq, counts[2, ]$Freq) out of c(226, 225)
## X-squared = 0.1101, df = 1, p-value = 0.74
## alternative hypothesis: two.sided
## 95 percent confidence interval:
##  -0.1112011  0.0726957
## sample estimates:
##    prop 1    prop 2 
## 0.3318584 0.3511111
```

```
prop.test(x = c(counts[6,]$Freq, counts[3,]$Freq), n = c(226, 225))
```

```
## 
##  2-sample test for equality of proportions with continuity correction
## 
## data:  c(counts[6, ]$Freq, counts[3, ]$Freq) out of c(226, 225)
## X-squared = 0.13256, df = 1, p-value = 0.7158
## alternative hypothesis: two.sided
## 95 percent confidence interval:
##  -0.07113358  0.11247085
## sample estimates:
##    prop 1    prop 2 
## 0.3495575 0.3288889
```

```
percents <- as.data.frame(prop.table(table(data$EVAL_ETHICAL, data$CONDITION), margin = 2))
ggbarplot(
  data = percents,
  x = "Var2",
  y = "Freq",
  title = "More ethical",
  fill = "Var1",
  position = position_stack()
) + scale_fill_manual(
  labels = c(
    "1" = "No code",
    "2" = "Show code",
    "3" = "Neither"
  ),
  values = c("red", "blue", "grey")
)
```

# 4 Choice

```
chisq.test(table(data$CHOICE, data$CONDITION))
```

```
## 
##  Pearson's Chi-squared test with Yates' continuity correction
## 
## data:  table(data$CHOICE, data$CONDITION)
## X-squared = 4.938, df = 1, p-value = 0.02627
```

```
counts <- as.data.frame(table(data$CHOICE, data$CONDITION))
prop.test(x = c(counts[3,]$Freq, counts[1,]$Freq), n = c(226, 225))
```

```
## 
##  2-sample test for equality of proportions with continuity correction
## 
## data:  c(counts[3, ]$Freq, counts[1, ]$Freq) out of c(226, 225)
## X-squared = 4.938, df = 1, p-value = 0.02627
## alternative hypothesis: two.sided
## 95 percent confidence interval:
##  0.01238942 0.19728610
## sample estimates:
##    prop 1    prop 2 
## 0.4115044 0.3066667
```

```
prop.test(x = c(counts[4,]$Freq, counts[2,]$Freq), n = c(226, 225))
```

```
## 
##  2-sample test for equality of proportions with continuity correction
## 
## data:  c(counts[4, ]$Freq, counts[2, ]$Freq) out of c(226, 225)
## X-squared = 4.938, df = 1, p-value = 0.02627
## alternative hypothesis: two.sided
## 95 percent confidence interval:
##  -0.19728610 -0.01238942
## sample estimates:
##    prop 1    prop 2 
## 0.5884956 0.6933333
```

```
percents <- as.data.frame(prop.table(table(data$CHOICE, data$CONDITION), margin = 2))
ggbarplot(
  data = counts,
  x = "Var2",
  y = "Freq",
  title = "Choice",
  fill = "Var1",
  position = position_dodge(0.8)
) + scale_fill_manual(
  labels = c(
    "1" = "No code",
    "2" = "Show code",
    "3" = "Neither"
  ),
  values = c("red", "blue", "grey")
)
```

# 5 Links between perveiving an option as ethical, and choosing it

```
table(data$EVAL_ETHICAL, data$CHOICE)
```

```
##    
##       1   2
##   1 107  37
##   2   5 149
##   3  50 103
```

```
chisq.test(table(data$EVAL_ETHICAL, data$CHOICE))
```

```
## 
##  Pearson's Chi-squared test
## 
## data:  table(data$EVAL_ETHICAL, data$CHOICE)
## X-squared = 164.3, df = 2, p-value < 2.2e-16
```
