# Supplementary material for: Public preferences regarding slow codes in critical care
Source: Bioethics. 2024 Oct 3;39(4):337–42. doi: 10.1111/bioe.13359 (PMC12015798; doi:10.1111/bioe.13359)
Supplement: Supplementary file 1 — Supporting information. [file BIOE-39-337-s001.zip › osfstorage-archive.zip!/Experiment 1/questionnaire.pdf]

Fragebogen

1 Intro

Trigger:

Zufallstrigger - Group - Bedingung: - Ausführungsposition: "direkt"
c_0001 = BALANCED RAND (1 , 3)

Welcome!

This survey is conducted by researchers of the University of Bamberg (Germany). Participation will take about 2 minutes. You will be asked to carefully read a short text about a situation in a hospital and answer a few questions about it.

All of your answers will be anonymized and used for scientific purposes only. Participation is voluntary and can be stopped at any time. In case of questions, please contact philipp.sprengholz@uni-bamberg.de.

Click next if you agree with the above terms and want to start the survey.

2 Scenario

Please read the following text very carefully. Take your time, there is no need to rush.

Mr. Johnson is a 70-year-old man with a history of heart disease and diabetes. He has been admitted to the ICU due to a severe heart attack, which has left him in a critical condition. Mr. Johnson is struggling to breathe. The medical team has conducted a thorough assessment and determined that his heart function is critically compromised, and there's minimal chance of a meaningful recovery. However, his family requests that everything possible is done to prolong his life.

The medical team decides for a "no code". This means that they do not use aggressive resuscitation measures and instead focus on palliative care to reduce Mr Johnson's pain as much as possible. This decision allows Mr Johnson to spend his remaining time peacefully with his family.

Please read the following text very carefully. Take your time, there is no need to rush.

Mr. Johnson is a 70-year-old man with a history of heart disease and diabetes. He has been admitted to the ICU due to a severe heart attack, which has left him in a critical condition. Mr. Johnson is struggling to breathe. The medical team has conducted a thorough assessment and determined that his heart function is critically compromised, and there's minimal chance of a meaningful recovery. However, his family requests that everything possible is done to prolong his life.

The medical team decides for a "full code". This means that they use all available and aggressive resuscitation measures, including intubation, defibrillation and drugs, in an attempt to revive Mr Johnson. This will be painful for Mr Johnson and, in the likely event that he does not recover, it also means that he will not be able to spend his remaining time peacefully with his family.

Please read the following text very carefully. Take your time, there is no need to rush.

Mr. Johnson is a 70-year-old man with a history of heart disease and diabetes. He has been admitted to the ICU due to a severe heart attack, which has left him in a critical condition. Mr. Johnson is struggling to breathe. The medical team has conducted a thorough assessment and determined that his heart function is critically compromised, and there's minimal chance of a meaningful recovery. However, his family requests that everything possible is done to prolong his life.

The medical team decides for a “slow code”. This means that they carry out resuscitation efforts, but at a slower pace or with less intensity, recognising that Mr Johnson's condition is unlikely to improve. This approach honours the family's wishes while recognising medical reality and avoiding pain for Mr Johnson in his remaining time.

How much do you agree or disagree with the following statements?

	strongly disagree						strongly agree
The medical team's decision is ethical.	◯	◯	◯	◯	◯	◯	◯
The medical team's decision is in Mr Johnson's best interests.	◯	◯	◯	◯	◯	◯	◯
The medical team's decision is in the best interests of Mr Johnson's family.	◯	◯	◯	◯	◯	◯	◯
The medical team's behavior is honest.	◯	◯	◯	◯	◯	◯	◯
The medical team should be punished for their decision.	◯	◯	◯	◯	◯	◯	◯

How often do you think medical teams make the decision described above when faced with similar situations?

◯ very seldom

◯

◯

◯

◯

◯

◯ very often

What did the medical team decide for?

- ◯ no code
- ◯ full code
- ◯ slow code

3 Endseite

Thank you for your participation! Please click on the link below to return to the CONNECT platform:

https://connect.cloudresearch.com/participant/project/994206d3ae8440bb8aea125fd4b7feaf/complete
