# Supplementary material for: Public preferences regarding slow codes in critical care
Source: Bioethics. 2024 Oct 3;39(4):337–42. doi: 10.1111/bioe.13359 (PMC12015798; doi:10.1111/bioe.13359)
Supplement: Supplementary file 1 — Supporting information. [file BIOE-39-337-s001.zip › osfstorage-archive.zip!/Experiment 1/analysis_output.html]

Show Code Experiment


# Show Code Experiment

```
library(ggpubr)
```

```
## Loading required package: ggplot2
```

```
library(sjPlot)
library(ggsignif)
library(dplyr)
```

```
## 
## Attaching package: 'dplyr'
```

```
## The following objects are masked from 'package:stats':
## 
##     filter, lag
```

```
## The following objects are masked from 'package:base':
## 
##     intersect, setdiff, setequal, union
```

```
#platform_data <- read.csv("assignments.csv")
#data <- readxl::read_xlsx("data.xlsx")
#data$ParticipantId <- data$p_0001
#data[data == -77] <- NA
#data <- merge(data, platform_data, by = "ParticipantId", all.x = TRUE)
#writexl::write_xlsx(data, "data_experiment_1.xlsx")

data <- readxl::read_xlsx("data_experiment_1.xlsx")

data$CONDITION <- ifelse(data$c_0001 == 1, "no code", ifelse(data$c_0001 == 2, "full code", "show code"))

table(data$CONDITION)
```

```
## 
## full code   no code show code 
##       148       151       151
```

# 1 Demographics

```
psych::describe(data$Age)
```

```
##    vars   n  mean    sd median trimmed  mad min max range skew kurtosis   se
## X1    1 450 45.68 15.81     46   45.16 21.5  18  80    62  0.2    -1.15 0.75
```

# 2 Evaluation of the codes

```
#ggviolin(
#  data = data,
#  x = "CONDITION",
#  y = "EVAL_HONEST",
#  add = "mean_ci",
#  trim = TRUE
#) + geom_signif(
#  comparisons = list(c("show code", "no code"), c("show code", "full code"), c("full code", "no code")), 
#  map_signif_level=TRUE,
#  step_increase = 0.1
#) 

plot_patient <- ggviolin(
  data = data,
  x = "CONDITION",
  y = "EVAL_PATIENT",
  add = "mean_ci",
  trim = TRUE,
  fill = "CONDITION",
  color = "white",
  add.params = list(color = "black")
) + geom_signif(
  comparisons = list(c("show code", "no code"), c("show code", "full code"), c("full code", "no code")), 
  map_signif_level=TRUE,
  step_increase = 0.1
) + coord_cartesian(
  ylim = c(1,7)
) + scale_y_continuous(
  expand = c(0,0),
  name = "Agreement with code\nbeing in patient's interest"
) + scale_x_discrete(
  name = "",
  labels = c(
    "no code" = "No\ncode",
    "show code" = "Slow\ncode",
    "full code" = "Full\ncode"
  )
) + scale_fill_manual(
  guide = FALSE,
  values = c(
    "no code" = "#dddddd",
    "show code" = "#FBA5A5",
    "full code" = "#a5a5fb"
  )
)

plot_family <- ggviolin(
  data = data,
  x = "CONDITION",
  y = "EVAL_FAMILY",
  add = "mean_ci",
  trim = TRUE,
  fill = "CONDITION",
  color = "white",
  add.params = list(color = "black")
) + geom_signif(
  comparisons = list(c("show code", "no code"), c("show code", "full code"), c("full code", "no code")), 
  map_signif_level=TRUE,
  step_increase = 0.1
) + coord_cartesian(
  ylim = c(1,7)
) + scale_y_continuous(
  expand = c(0,0),
  name = "Agreement with code\nbeing in family's interest"
) + scale_x_discrete(
  name = "",
  labels = c(
    "no code" = "No\ncode",
    "show code" = "Slow\ncode",
    "full code" = "Full\ncode"
  )
) + scale_fill_manual(
  guide = FALSE,
  values = c(
    "no code" = "#dddddd",
    "show code" = "#FBA5A5",
    "full code" = "#a5a5fb"
  )
)

plot_ethical <- ggviolin(
  data = data,
  x = "CONDITION",
  y = "EVAL_ETHICAL",
  add = "mean_ci",
  trim = TRUE,
  fill = "CONDITION",
  color = "white",
  add.params = list(color = "black")
) + geom_signif(
  comparisons = list(c("show code", "no code"), c("show code", "full code"), c("full code", "no code")), 
  map_signif_level=TRUE,
  step_increase = 0.1
) + coord_cartesian(
  ylim = c(1,7)
) + scale_y_continuous(
  expand = c(0,0),
  name = "Agreement with code\nbeing ethical"
) + scale_x_discrete(
  name = "",
  labels = c(
    "no code" = "No\ncode",
    "show code" = "Slow\ncode",
    "full code" = "Full\ncode"
  )
) + scale_fill_manual(
  guide = FALSE,
  values = c(
    "no code" = "#dddddd",
    "show code" = "#FBA5A5",
    "full code" = "#a5a5fb"
  )
)

plot_punish <- ggviolin(
  data = data,
  x = "CONDITION",
  y = "EVAL_PUNISH",
  add = "mean_ci",
  trim = TRUE,
  fill = "#dddddd",
  color = "white",
  add.params = list(color = "black")
) + geom_signif(
  comparisons = list(c("show code", "no code"), c("show code", "full code"), c("full code", "no code")), 
  map_signif_level=TRUE,
  step_increase = 0.1
) 


fig_1 <- ggarrange(
  plot_patient,
  plot_family,
  plot_ethical,
  ncol = 3,
  align = "hv"
)
```

```
## Warning: The `guide` argument in `scale_*()` cannot be `FALSE`. This was deprecated in
## ggplot2 3.3.4.
## ℹ Please use "none" instead.
## ℹ The deprecated feature was likely used in the ggpubr package.
##   Please report the issue at <https://github.com/kassambara/ggpubr/issues>.
## This warning is displayed once every 8 hours.
## Call `lifecycle::last_lifecycle_warnings()` to see where this warning was
## generated.
```

```
png("fig_1.png",height=8,width=22,units="cm",res=300)
print(fig_1)

fig_1
```

## 2.1 Patient interest

```
data %>% group_by(CONDITION) %>% summarise(
  mean(EVAL_PATIENT),
  sd(EVAL_PATIENT)
)
```

```
## # A tibble: 3 × 3
##   CONDITION `mean(EVAL_PATIENT)` `sd(EVAL_PATIENT)`
##   <chr>                    <dbl>              <dbl>
## 1 full code                 4.06               1.92
## 2 no code                   4.93               1.85
## 3 show code                 4.82               1.91
```

```
rstatix::cohens_d(EVAL_PATIENT ~ CONDITION, data = data)
```

```
## # A tibble: 3 × 7
##   .y.          group1    group2    effsize    n1    n2 magnitude 
## * <chr>        <chr>     <chr>       <dbl> <int> <int> <ord>     
## 1 EVAL_PATIENT full code no code   -0.462    148   151 small     
## 2 EVAL_PATIENT full code show code -0.396    148   151 small     
## 3 EVAL_PATIENT no code   show code  0.0598   151   151 negligible
```

```
t.test(data[data$CONDITION == "full code",]$EVAL_PATIENT, data[data$CONDITION == "no code",]$EVAL_PATIENT)
```

```
## 
##  Welch Two Sample t-test
## 
## data:  data[data$CONDITION == "full code", ]$EVAL_PATIENT and data[data$CONDITION == "no code", ]$EVAL_PATIENT
## t = -3.9938, df = 296.02, p-value = 8.21e-05
## alternative hypothesis: true difference in means is not equal to 0
## 95 percent confidence interval:
##  -1.3031335 -0.4427945
## sample estimates:
## mean of x mean of y 
##  4.060811  4.933775
```

```
t.test(data[data$CONDITION == "full code",]$EVAL_PATIENT, data[data$CONDITION == "show code",]$EVAL_PATIENT)
```

```
## 
##  Welch Two Sample t-test
## 
## data:  data[data$CONDITION == "full code", ]$EVAL_PATIENT and data[data$CONDITION == "show code", ]$EVAL_PATIENT
## t = -3.4271, df = 296.79, p-value = 0.0006962
## alternative hypothesis: true difference in means is not equal to 0
## 95 percent confidence interval:
##  -1.1970233 -0.3237391
## sample estimates:
## mean of x mean of y 
##  4.060811  4.821192
```

```
t.test(data[data$CONDITION == "no code",]$EVAL_PATIENT, data[data$CONDITION == "show code",]$EVAL_PATIENT)
```

```
## 
##  Welch Two Sample t-test
## 
## data:  data[data$CONDITION == "no code", ]$EVAL_PATIENT and data[data$CONDITION == "show code", ]$EVAL_PATIENT
## t = 0.51956, df = 299.71, p-value = 0.6038
## alternative hypothesis: true difference in means is not equal to 0
## 95 percent confidence interval:
##  -0.3138416  0.5390072
## sample estimates:
## mean of x mean of y 
##  4.933775  4.821192
```

## 2.2 Family interest

```
data %>% group_by(CONDITION) %>% summarise(
  mean(EVAL_FAMILY),
  sd(EVAL_FAMILY)
)
```

```
## # A tibble: 3 × 3
##   CONDITION `mean(EVAL_FAMILY)` `sd(EVAL_FAMILY)`
##   <chr>                   <dbl>             <dbl>
## 1 full code                5.74              1.44
## 2 no code                  4.07              2.24
## 3 show code                4.37              2.02
```

```
rstatix::cohens_d(EVAL_FAMILY ~ CONDITION, data = data)
```

```
## # A tibble: 3 × 7
##   .y.         group1    group2    effsize    n1    n2 magnitude 
## * <chr>       <chr>     <chr>       <dbl> <int> <int> <ord>     
## 1 EVAL_FAMILY full code no code     0.886   148   151 large     
## 2 EVAL_FAMILY full code show code   0.779   148   151 moderate  
## 3 EVAL_FAMILY no code   show code  -0.143   151   151 negligible
```

```
t.test(data[data$CONDITION == "full code",]$EVAL_FAMILY, data[data$CONDITION == "no code",]$EVAL_FAMILY)
```

```
## 
##  Welch Two Sample t-test
## 
## data:  data[data$CONDITION == "full code", ]$EVAL_FAMILY and data[data$CONDITION == "no code", ]$EVAL_FAMILY
## t = 7.6751, df = 257, p-value = 3.444e-13
## alternative hypothesis: true difference in means is not equal to 0
## 95 percent confidence interval:
##  1.241717 2.098806
## sample estimates:
## mean of x mean of y 
##  5.736486  4.066225
```

```
t.test(data[data$CONDITION == "full code",]$EVAL_FAMILY, data[data$CONDITION == "show code",]$EVAL_FAMILY)
```

```
## 
##  Welch Two Sample t-test
## 
## data:  data[data$CONDITION == "full code", ]$EVAL_FAMILY and data[data$CONDITION == "show code", ]$EVAL_FAMILY
## t = 6.7447, df = 272.16, p-value = 9.177e-11
## alternative hypothesis: true difference in means is not equal to 0
## 95 percent confidence interval:
##  0.967010 1.764241
## sample estimates:
## mean of x mean of y 
##  5.736486  4.370861
```

```
t.test(data[data$CONDITION == "no code",]$EVAL_FAMILY, data[data$CONDITION == "show code",]$EVAL_FAMILY)
```

```
## 
##  Welch Two Sample t-test
## 
## data:  data[data$CONDITION == "no code", ]$EVAL_FAMILY and data[data$CONDITION == "show code", ]$EVAL_FAMILY
## t = -1.242, df = 296.68, p-value = 0.2152
## alternative hypothesis: true difference in means is not equal to 0
## 95 percent confidence interval:
##  -0.7873261  0.1780546
## sample estimates:
## mean of x mean of y 
##  4.066225  4.370861
```

## 2.3 Ethicality

```
data %>% group_by(CONDITION) %>% summarise(
  mean(EVAL_ETHICAL),
  sd(EVAL_ETHICAL)
)
```

```
## # A tibble: 3 × 3
##   CONDITION `mean(EVAL_ETHICAL)` `sd(EVAL_ETHICAL)`
##   <chr>                    <dbl>              <dbl>
## 1 full code                 5.29               1.52
## 2 no code                   4.48               2.15
## 3 show code                 4.73               1.99
```

```
rstatix::cohens_d(EVAL_ETHICAL ~ CONDITION, data = data)
```

```
## # A tibble: 3 × 7
##   .y.          group1    group2    effsize    n1    n2 magnitude 
## * <chr>        <chr>     <chr>       <dbl> <int> <int> <ord>     
## 1 EVAL_ETHICAL full code no code     0.437   148   151 small     
## 2 EVAL_ETHICAL full code show code   0.317   148   151 small     
## 3 EVAL_ETHICAL no code   show code  -0.121   151   151 negligible
```

```
t.test(data[data$CONDITION == "full code",]$EVAL_ETHICAL, data[data$CONDITION == "no code",]$EVAL_ETHICAL)
```

```
## 
##  Welch Two Sample t-test
## 
## data:  data[data$CONDITION == "full code", ]$EVAL_ETHICAL and data[data$CONDITION == "no code", ]$EVAL_ETHICAL
## t = 3.7826, df = 270.42, p-value = 0.0001911
## alternative hypothesis: true difference in means is not equal to 0
## 95 percent confidence interval:
##  0.3901959 1.2372428
## sample estimates:
## mean of x mean of y 
##  5.290541  4.476821
```

```
t.test(data[data$CONDITION == "full code",]$EVAL_ETHICAL, data[data$CONDITION == "show code",]$EVAL_ETHICAL)
```

```
## 
##  Welch Two Sample t-test
## 
## data:  data[data$CONDITION == "full code", ]$EVAL_ETHICAL and data[data$CONDITION == "show code", ]$EVAL_ETHICAL
## t = 2.7468, df = 280.53, p-value = 0.006407
## alternative hypothesis: true difference in means is not equal to 0
## 95 percent confidence interval:
##  0.1592727 0.9648547
## sample estimates:
## mean of x mean of y 
##  5.290541  4.728477
```

```
t.test(data[data$CONDITION == "no code",]$EVAL_ETHICAL, data[data$CONDITION == "show code",]$EVAL_ETHICAL)
```

```
## 
##  Welch Two Sample t-test
## 
## data:  data[data$CONDITION == "no code", ]$EVAL_ETHICAL and data[data$CONDITION == "show code", ]$EVAL_ETHICAL
## t = -1.0555, df = 298.21, p-value = 0.292
## alternative hypothesis: true difference in means is not equal to 0
## 95 percent confidence interval:
##  -0.7208577  0.2175464
## sample estimates:
## mean of x mean of y 
##  4.476821  4.728477
```

# 3 Ethicality ratings by age and gender

```
model_nocode <- lm(EVAL_ETHICAL ~ Age + Gender, data[data$CONDITION == "no code",])
model_fullcode <- lm(EVAL_ETHICAL ~ Age + Gender, data[data$CONDITION == "full code",])
model_showcode <- lm(EVAL_ETHICAL ~ Age + Gender, data[data$CONDITION == "show code",])

tab_model(
  model_nocode,
  model_showcode,
  model_fullcode,
  show.se = TRUE,
  dv.labels = c("Ethicality of no code", "Ethicality of show code", "Ethicality of full code")
)
```

|  | Ethicality of no code | | | | Ethicality of show code | | | | Ethicality of full code | | | |
| --- | --- | --- | --- | --- | --- | --- | --- | --- | --- | --- | --- | --- |
| Predictors | Estimates | std. Error | CI | p | Estimates | std. Error | CI | p | Estimates | std. Error | CI | p |
| (Intercept) | 5.99 | 0.57 | 4.86 – 7.12 | **<0.001** | 5.32 | 0.50 | 4.34 – 6.30 | **<0.001** | 4.74 | 0.37 | 4.01 – 5.48 | **<0.001** |
| Age | -0.03 | 0.01 | -0.05 – -0.01 | **0.008** | -0.01 | 0.01 | -0.03 – 0.01 | 0.264 | 0.02 | 0.01 | 0.01 – 0.04 | **0.009** |
| Gender [Woman] | -0.10 | 0.35 | -0.79 – 0.59 | 0.777 | -0.18 | 0.33 | -0.83 – 0.46 | 0.579 | -0.61 | 0.25 | -1.10 – -0.12 | **0.014** |
| Observations | 151 | | | | 151 | | | | 148 | | | |
| R^2^ / R^2^ adjusted | 0.049 / 0.037 | | | | 0.011 / -0.002 | | | | 0.075 / 0.062 | | | |

# 4 Ethicality as a result of acting in the patient’s and the family’s best interest

```
cor.test(data$EVAL_ETHICAL, data$EVAL_HONEST)
```

```
## 
##  Pearson's product-moment correlation
## 
## data:  data$EVAL_ETHICAL and data$EVAL_HONEST
## t = 25.077, df = 448, p-value < 2.2e-16
## alternative hypothesis: true correlation is not equal to 0
## 95 percent confidence interval:
##  0.7228054 0.8001039
## sample estimates:
##       cor 
## 0.7641849
```

```
model <- lm(EVAL_ETHICAL ~ EVAL_PATIENT + EVAL_FAMILY, data = data)
tab_model(model, show.se=TRUE)
```

|  | EVAL\_ETHICAL | | | |
| --- | --- | --- | --- | --- |
| Predictors | Estimates | std. Error | CI | p |
| (Intercept) | 0.81 | 0.17 | 0.47 – 1.15 | **<0.001** |
| EVAL PATIENT | 0.37 | 0.03 | 0.30 – 0.44 | **<0.001** |
| EVAL FAMILY | 0.49 | 0.03 | 0.43 – 0.55 | **<0.001** |
| Observations | 450 | | | |
| R^2^ / R^2^ adjusted | 0.580 / 0.578 | | | |

# 5 Punishment

## 5.1 Group differences

```
data %>% group_by(CONDITION) %>% summarise(
  mean(EVAL_PUNISH),
  sd(EVAL_PUNISH)
)
```

```
## # A tibble: 3 × 3
##   CONDITION `mean(EVAL_PUNISH)` `sd(EVAL_PUNISH)`
##   <chr>                   <dbl>             <dbl>
## 1 full code                1.78              1.35
## 2 no code                  3.07              2.21
## 3 show code                2.89              1.90
```

```
rstatix::cohens_d(EVAL_PUNISH ~ CONDITION, data = data)
```

```
## # A tibble: 3 × 7
##   .y.         group1    group2    effsize    n1    n2 magnitude 
## * <chr>       <chr>     <chr>       <dbl> <int> <int> <ord>     
## 1 EVAL_PUNISH full code no code   -0.702    148   151 moderate  
## 2 EVAL_PUNISH full code show code -0.669    148   151 moderate  
## 3 EVAL_PUNISH no code   show code  0.0868   151   151 negligible
```

```
t.test(data[data$CONDITION == "full code",]$EVAL_PUNISH, data[data$CONDITION == "no code",]$EVAL_PUNISH)
```

```
## 
##  Welch Two Sample t-test
## 
## data:  data[data$CONDITION == "full code", ]$EVAL_PUNISH and data[data$CONDITION == "no code", ]$EVAL_PUNISH
## t = -6.081, df = 249.21, p-value = 4.463e-09
## alternative hypothesis: true difference in means is not equal to 0
## 95 percent confidence interval:
##  -1.6978044 -0.8670784
## sample estimates:
## mean of x mean of y 
##  1.783784  3.066225
```

```
t.test(data[data$CONDITION == "full code",]$EVAL_PUNISH, data[data$CONDITION == "show code",]$EVAL_PUNISH)
```

```
## 
##  Welch Two Sample t-test
## 
## data:  data[data$CONDITION == "full code", ]$EVAL_PUNISH and data[data$CONDITION == "show code", ]$EVAL_PUNISH
## t = -5.7963, df = 270.55, p-value = 1.884e-08
## alternative hypothesis: true difference in means is not equal to 0
## 95 percent confidence interval:
##  -1.4784944 -0.7287724
## sample estimates:
## mean of x mean of y 
##  1.783784  2.887417
```

```
t.test(data[data$CONDITION == "no code",]$EVAL_PUNISH, data[data$CONDITION == "show code",]$EVAL_PUNISH)
```

```
## 
##  Welch Two Sample t-test
## 
## data:  data[data$CONDITION == "no code", ]$EVAL_PUNISH and data[data$CONDITION == "show code", ]$EVAL_PUNISH
## t = 0.75441, df = 293.71, p-value = 0.4512
## alternative hypothesis: true difference in means is not equal to 0
## 95 percent confidence interval:
##  -0.2876615  0.6452774
## sample estimates:
## mean of x mean of y 
##  3.066225  2.887417
```

## 5.2 Differences by code and perceived ethicality

```
data$CONDITION_F <- factor(data$CONDITION, levels = c("no code", "show code", "full code"))

model <- lm(EVAL_PUNISH ~  EVAL_ETHICAL * CONDITION_F, data)
tab_model(model)
```

|  | EVAL\_PUNISH | | |
| --- | --- | --- | --- |
| Predictors | Estimates | CI | p |
| (Intercept) | 6.62 | 6.10 – 7.14 | **<0.001** |
| EVAL ETHICAL | -0.79 | -0.90 – -0.69 | **<0.001** |
| CONDITION F [show code] | -0.84 | -1.61 – -0.06 | **0.034** |
| CONDITION F [full code] | -3.68 | -4.64 – -2.71 | **<0.001** |
| EVAL ETHICAL × CONDITION F [show code] | 0.18 | 0.03 – 0.33 | **0.020** |
| EVAL ETHICAL × CONDITION F [full code] | 0.57 | 0.39 – 0.76 | **<0.001** |
| Observations | 450 | | |
| R^2^ / R^2^ adjusted | 0.487 / 0.481 | | |

```
fig_2 <- plot_model(
  model, 
  type = "int",
  alpha = 0.3
) + theme_classic(
) + coord_fixed(
  ylim = c(1,7)
) + scale_y_continuous(
  name = "Desire to punish medical team",
  breaks = c(1:7),
  expand = c(0,0)
) + scale_x_continuous(
  name = "Perceived ethicality of decision",
  breaks = c(1:7),
  expand = c(0,0)
) + ggtitle(
  ""
) + scale_fill_manual(
  name = "",
  values = c(
    "no code" = "#dddddd",
    "show code" = "#FBA5A5",
    "full code" = "#a5a5fb"
  ),
  labels = c(
    "no code" = "No code",
    "show code" = "Slow code",
    "full code" = "Full code"
  )
) + scale_color_manual(
  name = "",
  values = c(
    "no code" = "#dddddd",
    "show code" = "#FBA5A5",
    "full code" = "#a5a5fb"
  ),
  labels = c(
    "no code" = "No code",
    "show code" = "Slow code",
    "full code" = "Full code"
  )
) + theme(

      axis.ticks.x = element_line(color = "#000000", linetype = "solid", size=0.25),
      axis.ticks.y = element_line(color = "#000000", linetype = "solid", size=0.25),
      strip.text = element_text(color="#000000", size=12, angle=0),
      axis.text.y = element_text(color="#000000", size=12, angle=0),
      axis.title.y = element_text(color="#000000", size=12, angle=90),
      axis.title.x = element_text(color="#000000", size=12, angle=0),
      axis.text.x = element_text(color="#000000", size=12, angle=0),
      plot.title = element_text(color="#000000", size=12, angle=0, face="bold"),
      legend.text = element_text(color="#000000", size=12, angle=0),
      legend.title = element_text(color="#000000", size=12, angle=0),
      legend.position = "top"
  
) + guides(color=guide_legend(override.aes=list(fill=c(
    "no code" = "#dddddd",
    "show code" = "#FBA5A5",
    "full code" = "#a5a5fb"
  ))))
```

```
## Scale for y is already present.
## Adding another scale for y, which will replace the existing scale.
## Scale for colour is already present.
## Adding another scale for colour, which will replace the existing scale.
```

```
## Warning: The `size` argument of `element_line()` is deprecated as of ggplot2 3.4.0.
## ℹ Please use the `linewidth` argument instead.
## This warning is displayed once every 8 hours.
## Call `lifecycle::last_lifecycle_warnings()` to see where this warning was
## generated.
```

```
png("fig_2.png",height=11,width=12,units="cm",res=300)
print(fig_2)

fig_2
```

# 6 Estimated prevalence

```
ggviolin(
  data = data,
  x = "CONDITION",
  y = "PREVALENCE",
  add = "mean_ci",
  trim = TRUE
) + geom_signif(
  comparisons = list(c("show code", "no code"), c("show code", "full code"), c("full code", "no code")), 
  map_signif_level=TRUE,
  step_increase = 0.1
)
```

```
data %>% group_by(CONDITION) %>% summarise(
  mean(PREVALENCE),
  sd(PREVALENCE)
)
```

```
## # A tibble: 3 × 3
##   CONDITION `mean(PREVALENCE)` `sd(PREVALENCE)`
##   <chr>                  <dbl>            <dbl>
## 1 full code               5.78             1.38
## 2 no code                 5.01             1.76
## 3 show code               4.87             1.53
```

```
rstatix::cohens_d(PREVALENCE ~ CONDITION, data = data)
```

```
## # A tibble: 3 × 7
##   .y.        group1    group2    effsize    n1    n2 magnitude 
## * <chr>      <chr>     <chr>       <dbl> <int> <int> <ord>     
## 1 PREVALENCE full code no code    0.487    148   151 small     
## 2 PREVALENCE full code show code  0.625    148   151 moderate  
## 3 PREVALENCE no code   show code  0.0843   151   151 negligible
```

```
t.test(data[data$CONDITION == "full code",]$PREVALENCE, data[data$CONDITION == "no code",]$PREVALENCE)
```

```
## 
##  Welch Two Sample t-test
## 
## data:  data[data$CONDITION == "full code", ]$PREVALENCE and data[data$CONDITION == "no code", ]$PREVALENCE
## t = 4.2162, df = 283.07, p-value = 3.347e-05
## alternative hypothesis: true difference in means is not equal to 0
## 95 percent confidence interval:
##  0.4108012 1.1302763
## sample estimates:
## mean of x mean of y 
##  5.783784  5.013245
```

```
t.test(data[data$CONDITION == "full code",]$PREVALENCE, data[data$CONDITION == "show code",]$PREVALENCE)
```

```
## 
##  Welch Two Sample t-test
## 
## data:  data[data$CONDITION == "full code", ]$PREVALENCE and data[data$CONDITION == "show code", ]$PREVALENCE
## t = 5.4064, df = 294.94, p-value = 1.329e-07
## alternative hypothesis: true difference in means is not equal to 0
## 95 percent confidence interval:
##  0.5784939 1.2407293
## sample estimates:
## mean of x mean of y 
##  5.783784  4.874172
```

```
t.test(data[data$CONDITION == "no code",]$PREVALENCE, data[data$CONDITION == "show code",]$PREVALENCE)
```

```
## 
##  Welch Two Sample t-test
## 
## data:  data[data$CONDITION == "no code", ]$PREVALENCE and data[data$CONDITION == "show code", ]$PREVALENCE
## t = 0.73245, df = 294.13, p-value = 0.4645
## alternative hypothesis: true difference in means is not equal to 0
## 95 percent confidence interval:
##  -0.2346114  0.5127571
## sample estimates:
## mean of x mean of y 
##  5.013245  4.874172
```
